# Lifestyle discussions facilitate self-management in RA: a qualitative study of patients’ perceptions

**DOI:** 10.1186/s41927-024-00433-3

**Published:** 2024-11-29

**Authors:** Klara Drake af Hagelsrum, Ingrid Larsson, Ann Bremander, Jon T. Einarsson, Elisabet Lindqvist, Elisabeth Mogard

**Affiliations:** 1 Section of Rheumatology, Department of Clinical Sciences Lund, Lund University, Lund, Sweden; 2 School of Health and Welfare, Halmstad University, Halmstad, Sweden; 3 Spenshult Research and Development Centre, Halmstad, Sweden; 4 Department of Regional Health Research, University of Southern Denmark, Odense, Denmark; 5 Danish Hospital for Rheumatic Diseases, University Hospital of Southern Denmark, Sønderborg, Denmark; 6 Department of Rheumatology, Skåne University Hospital, Lund, Sweden

**Keywords:** Alcohol, Arthritis, Cardiovascular disease risk management, Diet, Healthcare, Lifestyle, Motivation, Person-centred care, Physical activity, Self-management, Tobacco, Qualitative content analysis

## Abstract

**Background:**

Healthy lifestyle habits (regular physical activity, a healthy diet, no smoking and non-hazardous alcohol consumption) alongside pharmacological treatment can lower the risk of cardiovascular diseases and improve symptoms and quality of life in patients with rheumatoid arthritis (RA). Therefore, healthcare professionals in rheumatology care are urged to discuss lifestyle habits with all patients. The aim of this study was to explore patients’ perceptions of lifestyle discussions in early rheumatology care.

**Methods:**

Individual interviews were conducted with 20 patients with RA, 14 women and six men, aged 23 to 77 years, and with a mean disease duration of 2.4 years. All lifestyle discussions were performed during the first year with RA. A qualitative content analysis was performed.

**Results:**

An overarching theme emerged, exploring how patients with RA perceived lifestyle discussions as facilitating self-management. Three categories illustrated this: (1) *the usefulness of lifestyle discussions* depended on the individual patient’s preferences and prioritization for lifestyle support; (2) *the design of lifestyle discussions* should be based on a person-centred approach, incorporating personalized lifestyle information and providing structured and recurrent support; (3) *the outcomes of lifestyle discussions* should contribute to enhanced knowledge and motivation for making healthy lifestyle changes.

**Conclusion:**

Lifestyle discussions in early rheumatology care should, according to patients with RA, be based on a person-centred approach, be tailored to each patient’s preferences and needs, and have outcomes focusing on patient support for healthy lifestyle changes, all essential elements to facilitate self-management. The present findings can be used to guide the development and implementation of more person-centred lifestyle approaches targeted to facilitate lifestyle changes and benefit cardiovascular disease risk management in early rheumatology care.

## Introduction

Rheumatoid arthritis (RA) is a chronic inflammatory systemic disease with symptoms such as stiffness, swelling, and joint pain. Controlling and keeping the inflammation at a low level is an essential part of the treatment [1, 2]. A combination of pharmacological and non-pharmacological treatments can relieve symptoms, prevent joint damage, improve or normalize physical function, quality of life, and work capacity [3, 4]. Patients with RA have an increased risk of developing cardiovascular diseases [5], and mortality in RA is elevated compared with the general population [6]. In the European Alliance of Associations for Rheumatology (EULAR) recommendations for cardiovascular disease risk management, a risk assessment is recommended at least once every five years for all patients with RA. It is also recommended that healthcare professionals (HPs) provide advice and offer support for a healthy lifestyle at clinical visits, which potentially may lower the cardiovascular risk [3].

To support lifestyle management in rheumatology care, national and international guidelines focus primarily on lifestyle habits such as physical activity, dietary habits, smoking, and alcohol consumption [7, 8]. In patients with RA, positive cardiovascular effects have been observed after structured physical activity [9, 10]. Other benefits of physical activity include reports of increased self-esteem, reduced pain, relief of symptoms of depression, and improved sleep quality [11, 12]. An anti-inflammatory diet, such as a mediterranean diet, has been associated with a reduced incidence of cardiovascular diseases in the general population and is also recommended for patients with RA [3, 13, 14]. There are additional indications that an anti-inflammatory diet can have a positive effect on disease activity in patients with RA [15, 16]. Overweight or obese patients with RA have an increased risk of cardiovascular disease and other comorbidities, such as pain and physical disability [17]. Smoking increases the risk of developing RA, and smokers with RA may also have a more severe disease course [18], and an inferior effect from their drug treatment [19, 20]. It is known that alcohol can interact with anti-rheumatic drugs [2], and the effect has been suggested to depend on the dose and duration [13]. Alcohol consumption may also worsen symptoms, function, and disease activity, and increase disease progression and occurrence of comorbidities in people with RA [21]. However, previous research is not unequivocal regarding alcohol consumption and the influence on disease activity in RA [22].

Unhealthy lifestyle habits are common among patients with RA, with reports from a Scandinavian study showing that about half of the patients had two or more unhealthy lifestyle factors. The most common were low levels of physical activity and being overweight or obese [23]. Despite this, another study in patients with established RA showed that only half of the patients recalled having discussed physical activity with HPs, while a quarter or less recalled discussions on dietary habits, smoking, and alcohol consumption [24]. Most patients were aware of general health recommendations, but struggled with ‘a constant balancing between ideality and reality’, a struggle impacted by their RA and affecting their quality of life [25].

HPs are essential in the work to motivate patients to achieve a healthy lifestyle by spreading knowledge [26]. Therefore, patient education with preventive information, guidance, and support is important in lifestyle management [3, 27]. In a previous study of ours, HPs in rheumatology emphasized the importance of prioritizing and structuring team-based and patient-centred lifestyle management [28]. However, more knowledge is needed regarding lifestyle discussions and patient education from the patients’ perspective, and particularly on how such discussions can facilitate healthy lifestyle changes [3]. While previous research highlights the importance of lifestyle factors in RA management and cardiovascular risk reduction, there is still limited understanding of how patients perceive lifestyle discussions in clinical care. With this qualitative, explorative study we seek to address this gap by gathering novel insights that can enhance a person-centred approach and strengthen the dialogue between patients and HPs during lifestyle discussions.

Exploring patients perceptions on lifestyle management and the support needed for patients in early RA management to make healthier lifestyle choices could ultimately improve the support provided for patients in early RA management and facilitate healthy lifestyle changes. Therefore, the aim of this study was to explore patients’ perceptions of lifestyle discussions in early rheumatology care.

## Methods

### Design

This study has an exploratory design with latent qualitative content analysis and an inductive approach to understanding patients’ perceptions of lifestyle discussions. This non-linear process involves decontextualization and recontextualization [29]. The Consolidated Criteria for Reporting Qualitative Research 32-item checklist was used to ensure trustworthiness [30].

### Participants

The participants were recruited from a specialist rheumatology clinic in Sweden, where lifestyle discussions, as recommended by EULAR [3], are regularly performed in clinical meetings with HPs in the interdisciplinary team. During the first year with RA, patients frequently visit a rheumatologist and a rheumatology nurse, according to a tight control regimen. At some of those visits, the four lifestyle habits − physical activity, dietary habits, smoking, and alcohol consumption − are addressed, and a cardiovascular risk assessment is performed, according to a structured schedule. Eight weeks after the RA diagnosis, patients are also invited to a meeting with a physiotherapist and an occupational therapist where lifestyle habits, among other things, are discussed. The physiotherapist evaluates patients’ current physical activity levels and offers individual information and support to enhance physical activity when needed. The occupational therapist focuses on occupational balance, including lifestyle challenges. A social worker can be involved when needed, for example by offering support regarding lifestyle changes.

The inclusion criteria were patients diagnosed with RA, according to ACR/EULAR 2010 criteria [31], in 2019 or 2020 at a specialist rheumatology clinic, and who participated in lifestyle discussions during the first year after their RA diagnosis. Purposeful sampling was applied to obtain a variation in age and gender to capture variation in experiences [32]. A total of 24 patients were invited by a researcher at the clinic to participate in the study. Twenty patients accepted the invitation, comprising 14 women and six men aged 23 to 77 years, and a mean disease duration of 2.4 years (Table 1). Clinical characteristics for all patients are presented in Table 1.

**Table 1:** Patients’ characteristics, *n* = 20

Characteristics	
Sex, female, n (%)	14 (70)
Disease duration, mean (SD)	2.4 (0.5)
Anti-CCP positivity, n (%)	20 (100)
DAS28-CRP at closest visit, mean (SD)	2.5 (1.2)
NRS pain, mean (SD)	2.8 (2.4)
NRS global, mean (SD)	3.4 (2.6)
NRS fatigue, mean (SD)	4.1 (3.1)
PASS (acceptable, yes), n (%)	14 (88)
Physio- and occupational therapist consultation within three months from diagnosis, n (%)	17 (85)
Ongoing treatment, n (%)	20 (100)
csDMARDs	15 (75)
bDMARDs	8 (40)
tsDMARDs	5 (25)
Corticosteroids	0

Missing data: NRS and PASS (*n* = 4)

Abbreviations; SD; standard deviation, Anti-CCP; anti-Cyclic Citrullinated Peptide, DAS28-CRP; disease activity score using C-reactive protein, NRS; Numeric Rating Scales, PASS; Patient acceptable symptom state cs/b/tsDMARDs; synthetic/biologic/targeted disease-modifying anti-rheumatic drugs

### Data collection

Data collection with individual interviews was conducted between January 2022 and February 2023. The semi-structured interview guide with open-ended questions focused on how patients perceived discussions about lifestyle habits with HPs at a specialist rheumatology clinic. Questions asked included, for example: “How do you perceive lifestyle discussions in rheumatology care?”, “How would you like to discuss lifestyle habits with HPs?” and “What support do you need to change a lifestyle habit?” Probing questions were “Can you tell me more?”, “What do you have in mind when you say …?”. The interviews were conducted by three of the authors (KDaH, IL, AB), who had no previous relationship with the participants. The researchers IL and AB have documented experience in qualitative methods and rheumatology, and KDaH was under supervision from IL. All interviews were conducted by telephone and lasted between 30 and 97 minutes, with a total interview time of 15 hours and 30 minutes. The interviews were digitally recorded and transcribed verbatim.

### Data analysis

An inductive qualitative content analysis was performed [33, 34], and the transcribed interviews were read thoroughly several times, searching for meaning units describing patients’ perceptions of lifestyle discussions. A total of 485 meaning units were identified, condensed, abstracted, and coded. From the codes, similarities and differences were identified and sorted into seven sub-categories. The sub-categories were then sorted into three categories, representing the manifest content. For example, the sub-categories “person-centred approach” and “recurrent lifestyle support” were sorted into the category “The design of lifestyle discussions”. The underlying meaning of the categories, the latent content, was then formulated as an overarching theme. The first (KDaH) and second (IL) authors performed the analysis, with ongoing discussions within the multidisciplinary research group until a consensus was reached. The research group had extensive experience in rheumatology care and qualitative methods, and included registered nurses, physiotherapists, and rheumatologists.

## Results

The results revealed an overarching theme exploring how patients with early RA perceived that lifestyle discussions in rheumatology care could facilitate self-management. Three categories described how the usefulness, design, and outcomes of lifestyle discussions facilitated this self-management (Table 2). The usefulness of lifestyle discussions depended on patients’ personal preferences for lifestyle support and their prioritization to discuss lifestyle habits with HPs. The design of a lifestyle discussion aiming to improve adherence to healthy lifestyle habits should, according to the patients, be based on a person-centred approach, including individually tailored information and structured recurrent support. Finally, the outcomes of lifestyle discussions should improve knowledge and facilitate patients’ motivation to make healthy lifestyle changes.

**Table 2:** The overarching theme, categories, and sub-categories that describe patients’ perceptions of lifestyle discussions in early rheumatology care

Overarching theme	Lifestyle discussions in rheumatology care facilitate self-management		
Categories	**The usefulness of lifestyle discussions**	**The design of lifestyle discussions**	**The outcomes of lifestyle discussions**
Sub-categories	Personal needs and preferences for lifestyle support Prioritization of lifestyle discussions	A person-centred approach Personalized lifestyle information Structured and recurrent lifestyle support	Knowledge of healthy lifestyle habits Motivation for healthy lifestyle changes

## The usefulness of lifestyle discussions

The usefulness of lifestyle discussions among patients with early RA depended on their needs and preferences for lifestyle support and their prioritization of these discussions. Acknowledging each patient’s condition and general situation when discussing lifestyle habits was expressed as important. Lifestyle discussions were also experienced as more or less useful depending on the patients’ current motivation and priorities in their disease process; sometimes mastering flares with pain and fatigue was given higher priority.

### Personal needs and preferences for lifestyle support

Some patients preferred lifestyle support to improve their health, while others felt that lifestyle discussions were not useful for them and that it was not up to certain HPs to discuss lifestyle habits. It could depend on whether the disease was stable, if the patient experienced pain, had support from elsewhere, or sought out the information themselves, for example on-line. According to the patients, lifestyle discussions were more useful if they were focused on facilitating patients’ motivation to change their behaviour and encourage self-management. The usefulness also depended on the patient’s condition and general health situation, which could be challenging after receiving an RA diagnosis.

If there is something you can do that will make you feel better for several years to come, then you want to do it … Then I think that … if it’s something that is as essential as medication, then it’s important that they (the HPs) present that information as well. (Participant no. 11)

Patients described how lifestyle discussions with HPs in the interdisciplinary team contributed to different perspectives on lifestyle habits. It was suggested that lifestyle support through team meetings where a patient’s individual lifestyle habits were discussed should be offered. Patients also highlighted the usefulness of receiving information early in the disease course to facilitate self-management. Some patients experienced that HPs were genuinely interested in helping them improve their lifestyle habits, while some perceived discussions about healthy lifestyle habits as excessive or lacking structure.

I think that three-way talks are good. Different professions that meet the patient usually give more than individual meetings. (Participant no. 4)

### Prioritization of lifestyle discussions

Patients’ perceptions of the prioritization of lifestyle discussions during their meetings with HPs varied. Some patients perceived that they had engaged in valuable and extensive discussions concerning lifestyle habits with HPs at the rheumatology clinic, which they found to be useful for them.

They have been asking from the beginning, asking questions and so on. So, I feel that they want to find out things. (Participant no. 2)

Others stated that no discussions had taken place. Some patients perceived that lifestyle discussions were de-prioritized in favour of more urgent needs, for example, until the pharmacological treatment response was adequate, which also seemed reasonable, according to the patients.

I feel that my contact has been mostly about medications … so there has not been much focus on the other [e.g. lifestyle discussions]. (Participant no. 2)

## The design of lifestyle discussions

Patients with early RA perceived that lifestyle discussions should be based on a person-centred approach, personalized lifestyle information, and structured, recurrent lifestyle support to facilitate self-management.

### A person-centred approach

Patients expressed that person-centred discussions, focusing on their personal needs and disease conditions, enhanced motivation for healthy lifestyle changes. They also described a need for HPs to have a holistic view to provide self-management early in the disease course.

When talking it should not be generally, you should do this, you should do that, you should do the other, but instead focus on this is good for your joints. (Participant no. 15)

It was suggested that social workers and dieticians could play a more significant role in lifestyle discussions than currently offered, and that lifestyle discussions should include personal dietary advice and personalized exercise programs. Some patients also suggested supervised group exercises to strengthen motivation as part of the lifestyle discussions.

I think it’s fun to do exercises together with others. Then it becomes automatic that you might get tips and ideas in the classes, perhaps about how to eat or how to do something else … (Participant no. 20).

### Personalized lifestyle information

The design of lifestyle discussions should, according to the patients, include a two-way discussion and contain both general information about healthy lifestyle habits and more individually tailored information based on patients’ conditions and needs. Receiving relevant information from different HPs was necessary to facilitate self-management, but patients also wanted a personalized discussion rather than just general information. Personalized information, adapted to each patient’s previous knowledge, could also increase the motivation for a healthy lifestyle change.

General information feels like it just passes over you … but if it’s more personalized, you feel like, ‘okay, now someone has really looked at how I would probably be affected by this’, so perhaps you take more notice of it. (Participant no. 17)

Some patients valued information about lifestyle habits that are explicitly linked to RA. This could include, for example, information about the prognosis and risk of comorbidities of RA. Others expressed that detailed information about consequences could evoke less motivation to improve lifestyle habits and, on the contrary, induce a feeling of being able to perform less in everyday life activities.

I’ve never received that information, about chronic fatigue, but I’ve read that myself. But I’ve had periods when I’ve only gone up and out with the dog; yes, she gets her walks, and the rest of the time I’ve been sitting and sleeping on the sofa and couldn’t do anything … So information about what to expect when you get rheumatism, is probably what I miss. (Participant no. 1)

Written information was perceived as essential for remembering details about different healthy lifestyle habits, especially during periods of flares with increased pain and functional limitations, when such habits could be easily forgotten. Some patients stated that they had received written information concerning the impact of lifestyle habits when living with RA, while others did not. Some wanted more written information, while others were satisfied or expressed that written information ends up unread. Discussing lifestyle habits with HPs was perceived as the best way to convey relevant and personalized information about healthy lifestyle habits.

Unfortunately, paper and such tend to end up in the trash… when you’re there and talking to the nurse …that’s probably the easiest way to spread and retain it [the information]. (Participant no. 18)

### Structured and recurrent lifestyle support

The need for structured and recurrent support from HPs to facilitate self-management of lifestyle habits differed between patients. Some preferred structured follow-ups, while others expressed that it was up to them to contact, for example, the physiotherapist, regarding support on physical activity after the first meeting.

Then, that you really give feedback, ‘Do you have time to come in for a discussion?’. Then there is a little pressure on yourself, that you have to try, because it’s about me and my own best interests. So, I think follow-up is great. (Participant no. 8)

Most patients expressed that all lifestyle habits were addressed in some way in meetings with HPs, but not necessarily discussed. Other patients did not remember that lifestyle discussions had taken place, or at least not in a structured way, and this could depend on the fact that no structured follow-up with HPs had occurred.

Maybe it is the fact that there hasn’t been much follow-up, that there hasn’t been much focus on it (healthy lifestyle habits) later … that you have, sort of forgotten, because it was so long ago. (Participant no. 17)

It was perceived that lifestyle discussions sometimes ‘fizzled out’ and came to nothing. The large amount of information and the shock of receiving a rheumatic diagnosis could, according to the patients, also affect the sense of importance of the information regarding lifestyle habits. The patients suggested that the design should include follow-up meetings to concur with set goals, and that follow-up arrangements could be performed in different ways: by telephone, physical meetings, group sessions, but also include physiological tests. Recurring follow-up meetings were perceived as necessary since the disease changes over time.

Absolutely follow-ups, because I mean … things happen in your body with the disease and in life things can change, so yes, absolutely a follow-up. (Participant no. 16)

## The outcomes of lifestyle discussions

Patients perceived that increased knowledge about healthy lifestyle habits was an essential outcome of lifestyle discussions, and that this could increase motivation for healthy lifestyle changes and facilitate self-management.

### Knowledge of healthy lifestyle habits

Disease-specific information and increased knowledge about the advantages of healthy lifestyle habits in general were, according to the patients, important outcomes of lifestyle discussions and could facilitate self-management.

I still think that they have been very clear and helpful in general at the rheumatology clinic. I feel that I have been treated well. They have provided good information. (Participant no. 8)

Many patients appreciated the opportunity to discuss healthy lifestyle habits with various HPs in the interdisciplinary team; however, patients’ degree of satisfaction with lifestyle discussions differed. Some perceived that they did not get enough knowledge about the advantages of healthy lifestyle changes, while others considered that they already had healthy lifestyle habits or expressed no need for more knowledge.

It works well overall, for me. Because of that, I don’t think you need to spend more energy on it. (Participant no. 19)

### Motivation for healthy lifestyle changes

Motivation emerged as an essential outcome of lifestyle discussions and was perceived to support self-management. Patients expressed that motivation must come from within but could also be increased during a discussion with trusted HPs. According to the patients, relevant and clear information about lifestyle habits was necessary to create motivation.

I don’t think I was motivated enough to do it then. But maybe I could have been if I had received information. (Participant no. 13)

Some patients perceived that programs tailored to a specific lifestyle habit performed individually and in groups could increase motivation. Others expressed that they would be motivated by physical tests on follow-up visits or even follow-up telephone calls. Group sessions, with the opportunity to talk to other patients with the same diagnosis, were also found to be an excellent opportunity to increase motivation.

Then maybe with the physiotherapist you would have some type of physical tests … for example, I think I can handle more than I do, that I’m bad at listening to my body and taking a break when necessary. (Participant no. 17)

Discussions about healthy lifestyle habits were perceived to improve a sense of responsibility for their bodies and were central to creating motivation. Avoiding the consequences of inactivity, such as reduced energy, increased pain and stiffness, was described as a motivation for increasing physical activity. Improved physical activity and reduced sedentary time also had a mental effect and motivated patients to maintain lifestyle changes.

If I can exercise and feel stronger physically, then I also feel a little stronger mentally. (Participant no. 5)

## Discussion

Support to enhance positive lifestyle changes is an essential part of rheumatology care, alongside modern pharmacological treatment [7]. In this qualitative study, lifestyle discussions during the first year with RA were, according to patients, perceived to facilitate self-management. Patients described that HPs must consider each patient’s preferences and individual prioritization of lifestyle discussions to be perceived as useful, and that the design of lifestyle discussions should involve a person-centred approach, with personalized information, and structured, recurrent lifestyle support. In addition, the outcomes of lifestyle discussions should aim to increase a patients’ knowledge of potential positive or adverse effects of lifestyle habits on their RA and facilitate their motivation to strive towards a healthier lifestyle. Structured lifestyle discussions in early rheumatology care are thus one way to incorporate self-management into routine clinical care to a greater extent [35].

Personal preferences, such as each patient’s health condition, stage in the disease process, pharmacological treatment respons, general situation, and prior knowledge influenced how useful or prioritized the patients in this study found lifestyle discussions. This adheres to EULAR’s recommendations regarding lifestyle behaviours [7], where HPs in rheumatology care are urged to adapt lifestyle discussions and support to a patient’s current health condition and pharmacological treatment, but also taking into account different personal factors, including age and sex. In this study we cannot say if this also was influenced by important aspects such as social status, education or health literacy. A previous study in established RA found that more than half of the patients did not recall having discussed lifestyle habits such as physical activity or diet with HPs in rheumatology care, and that less than two out of 10 wanted to have those discussions. Recollections of discussions regarding smoking and alcohol were even fewer [24]. Whether patients in our study found lifestyle discussions useful or not was also influenced by their perceived needs for knowledge related to lifestyle habits, as some sought information themselves, for example on-line, or had support from elsewhere. In two other studies regarding educational needs in RA and other rheumatic diseases, five to six out of 10 patients reported not only a need for more information but also that the information be individually tailored, and this request was more common in patients with higher disease activity [36, 37].

The patients in the present study perceived that involvement from HPs with different professional expertise and RA-specific information related to different lifestyle habits was important when discussing healthy lifestyle habits. This complies with the prerequisites for person-centred care [38, 39] and previous studies in early RA, where interdisciplinary teams were described as beneficial when designing a person-centred care approach in lifestyle discussions [28, 40]. It also corresponds well with a qualitative study in general practice where patients expressed the importance of person-centred care when discussing lifestyle habits with HPs [41]. Patients should thus be included in the care process, confirmed as essential in previous research, according to patients themselves [42] and HPs in rheumatology care [28].

Personalized lifestyle information emerged as an essential part of lifestyle discussions to gain knowledge about healthy lifestyle habits in relation to RA. Patients expressed that the context of lifestyle discussions was important to consider and necessary for well-structured discussions. They also highlighted recurrent lifestyle support as essential but not always offered. In a previous study, HPs in rheumatology care also expressed the importance of recurrent lifestyle support and that this, due to resources and structure, was not always possible [28]. In supporting patients’ self-management, patient education should, according to EULAR recommendations, serve as the starting point and foundation throughout the disease process [43]. It should be tailored to an individual patient’s needs and integrated into standard care [27]. Patients suggest that education should be up-to-date, well-timed, and delivered by HPs with continuous training [44]. By providing insights from the patients’ perspectives, the current study may contribute to bridging the gap between real-life practice and best practice evidence.

In-depth knowledge of the relationship between lifestyle habits and their effects on the RA disease, together with the advantages of a healthy lifestyle in more general terms, were perceived as important outcomes of lifestyle discussions that could enhance patients’ motivation for healthy lifestyle changes and provide self-management. This complies with EULAR recommendations emphasizing that advice regarding healthy lifestyle habits is communicated by HPs with core competences in rheumatology and skills in communication and behaviour change techniques, e.g., motivational interviewing [45], and are evidence-based and a complement to pharmacological treatment to manage possible disease consequences and comorbidities [7].

In this study, patients suggested that lifestyle discussions incorporated into activities such as individually tailored exercise programs and group sessions, as well as meetings with other patients via activities organized by the rheumatology clinic, were essential activities that could contribute to increased motivation for healthy lifestyle changes. They also expressed that even though motivation for healthy lifestyle changes must come from within, person-centred lifestyle discussions and cooperation with trusted HPs could increase motivation to change behaviour. This aligns with the importance of individual and guided support, which considers a person’s barriers and abilities to manage the consequences of chronic disease, and holistically aims to strengthen self-management skills [43]. In addition, a systematic review regarding initiation and maintenance of behaviour change suggests that a major life or health event, such as a recent diagnosis of RA, can support initial behaviour change, while maintaining a habit requires active self-regulation, which in turn is dependent on e.g., personal resources, how strong a habit is, and social and environmental contexts [46]. The patients in this study described a feeling of responsibility for their bodies, e.g., by engaging in physical activities or eating healthy food, and expressed that this could create satisfaction and motivation for a healthier lifestyle. Previous research emphasizes that patients, in addition to information, need to feel in control of their disease [47], and that HPs in rheumatology care can facilitate patients’ confidence and self-management skills by encouraging patients to engage in shared responsibility and decision-making to manage their condition [43, 48]. It has also been emphasized that patients with RA want to feel empowered and regain daily activities, work, and leisure time activities, all of which are important to improve quality of life [49]. In this context, person-centred lifestyle discussions between patients and HPs emerged as an essential element for increasing self-management during the first years after an RA diagnosis. This study did not include questions regarding patients’ usage or thoughts on for example counselling apps, which could have been interesting to explore in future research since the usage of web-based applications and apps is increasing and some patients may have been obliged to use this.

To assess the trustworthiness of the study, strengths and limitations are discussed using the qualitative concepts of credibility, dependability, confirmability, and transferability [49]. The data covered variations in experiences of the phenomenon, and the rich and detailed descriptions of the methodological process strengthen the study’s credibility, which refers to confidence in the truth of the data and the analysis process [50]. Credibility was strengthened by including 20 patients, following the concept of ‘information power,’ which suggests that a smaller, focused sample can provide valuable insights, especially when the study aims are clear and the quality of dialogue is prioritized. This sample size allowed for an in-depth exploration of patients’ perceptions of lifestyle discussions in early rheumatology care, ensuring data saturation was reached, as no new sub-categories emerged after the 17th interview, and the information enriched the existing categories [51]. The interviews were based on an interview guide, ensuring that the same questions were asked of all participants, strengthening the study’s dependability [50]. It can be both a strength and a limitation that three researchers conducted the interviews. The researchers’ various professions were a strength, providing a broader understanding and variation of the phenomenon. However, the researchers’ preconceptions can influence the interpretation of the participants’ narratives and, thus, the follow-up questions. The interviewing researchers and the researcher analysing the data had no established relationship with the participants. Quotes have been used to reflect patients’ voices and show variations and similarities in the material, strengthening data neutrality and confirmability [50]. All participants were recruited from a single rheumatology university clinic, which may limit the study’s transferability to other populations and healthcare settings. However, since the clinic serves a large area of southern Sweden and adheres to international and national guidelines for lifestyle management, the study’s results may be transferable to other rheumatology clinics. To further strengthen the transferability, a purposeful selection of patients was used ensuring a variation in age and gender to capture a broad variation in experiences [33, 34].

## Conclusion

Structured and recurrent person-centred lifestyle discussions in early rheumatology care have the potential to facilitate self-management during the first years with RA, according to patients. They expressed that meaningful lifestyle discussions should be tailored to each patient’s personal preferences and needs and should focus on increased knowledge and personalized lifestyle support, which in turn was perceived to facilitate patients’ motivation to strive for healthy lifestyle changes. These findings can inform ongoing efforts to improve lifestyle management strategies and support patient self-management in early rheumatology care, which may ultimately contribute to reducing cardiovascular disease risk for patients with RA.

## Data Availability

Empirical material generated and/or analysed during the current study are not publicly available but are available from the corresponding author on reasonable request.
